# MUSIC IN THE LIFE COURSE: A FOCUS ON DEMENTIA

**DOI:** 10.1093/geroni/igz038.3101

**Published:** 2019-11-08

**Authors:** Amanda Couve, Joseph A Kotarba

**Affiliations:** 1 Encompass Health - Hospice, San Antonio, Texas, United States; 2 Texas State University, San Marcos, Texas, United States

## Abstract

The music experiences of Baby Boomers (born 1946 to 1965) continue to evolve as they age, with the Beatles and the Blues remaining key features. Baby boomers grew up in the post-World War II period when the variety of media technologies and outlets grew tremendously. Baby boomers continue this trend to experience and enjoy music through media such as theme cruises, community center activities, formal concerts, TV music awards programs, iPhones, and house concerts. The value of music in caring for the increasing number of Baby Boomers living with dementia is also increasing. For example, music can help patients recall pleasant and calming moments in their past, while helping enhance body movement and balance through dance. The audience’s’ takeaways will be ideas for creative marketing of music to the elderly; for integrating music into senior living situations, and for celebrating technology’s contributions to the culture of aging.

